# Supplementary material for: Replication confers β cell immaturity
Source: Nat Commun. 2018 Feb 2;9:485. doi: 10.1038/s41467-018-02939-0 (PMC5797102; doi:10.1038/s41467-018-02939-0)
Supplement: Supplementary file 1 — Supplementary Information [file 41467_2018_2939_MOESM1_ESM.pdf]

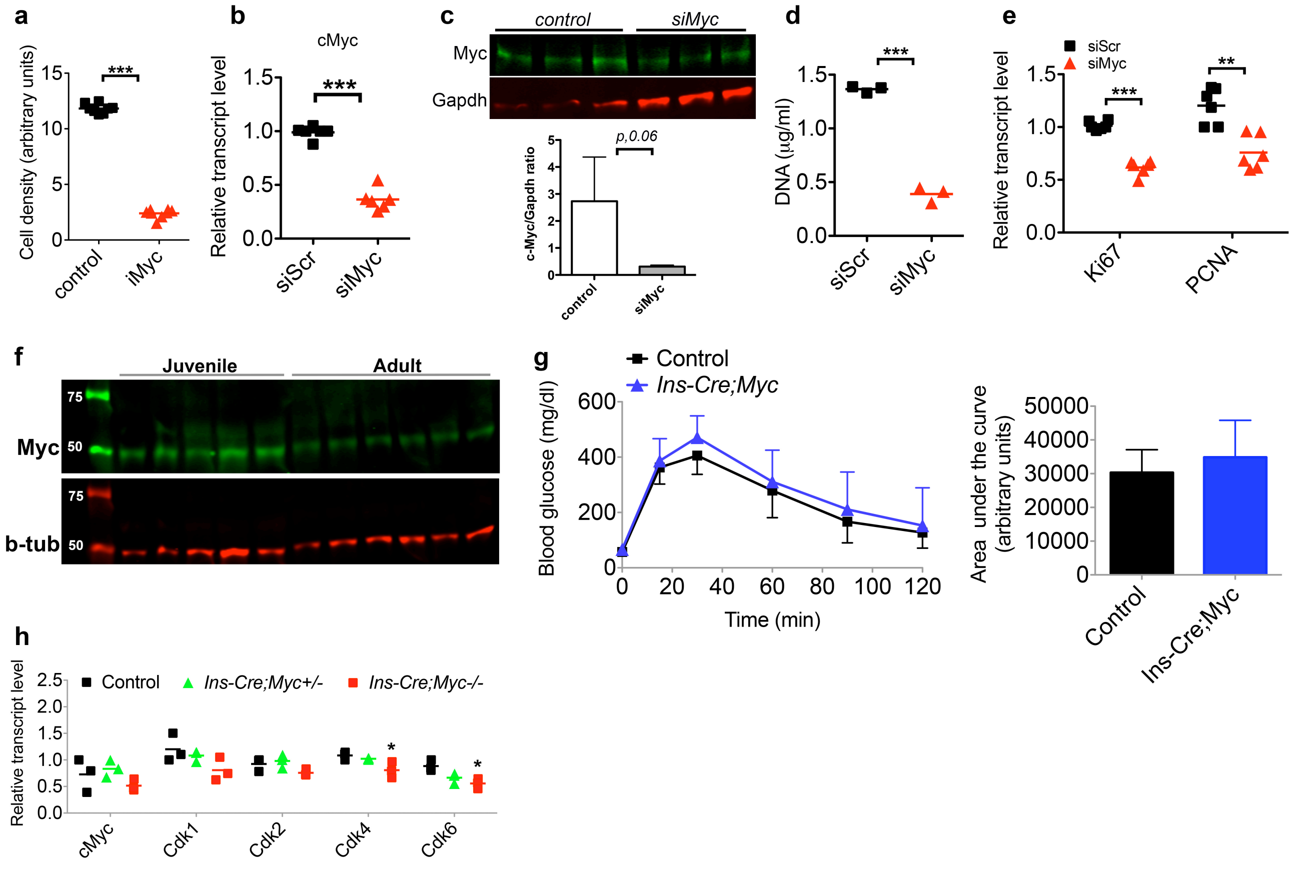

Supplementary Figure 1. **Inhibition of c-Myc activity.** **a**. INS-1 cells treated with Myci (40µM) show reduced cellular density after three days of treatment. n=8 for each condition. ****p*<0.0005, Student's *t* test. **b**. Transfection of INS-1 cell with siMyc for five days leads to a reduction in c-Myc expression by RNA quantification. n=6 per condition. ****p*<0.0005, Student's *t* test. **c**. siMyc transfection in INS-1 cells for five days leads to reduced c-Myc protein levels as compared to siScr. Gapdh was used as a housekeeping protein. n=3 per condition. Error bars indicate ± SD. *p*=0.06, Student's *t* test. **d**. siMyc transfection for five days leads to a reduction in cellular density in INS-1 cells. n=3 per condition. ****p*<0.0005, Student's *t* test. **e**. QPCR revealed reduced expression of *Ki67* and *Pcna* in siMyc-transfected samples after five days. n=6 per condition. ***p*<0.005, ****p*<0.0005, Student's *t* test. **f**. Western blotting shows reduced Myc expression in islets from adult (3 months old, n=6) versus juvenile (3 weeks old, n=5) wild type animals. Beta-tubulin was used as a loading control. **g**. Glucose challenge shows a trend towards mild glucose intolerance in 3-month-old animals lacking one or both alleles of c-Myc (*Ins-Cre;Myc*) as compared to controls. Error bars indicate ± SD. **h**. QPCR analysis indicates a trend towards loss of cell cycle regulators in islets isolated from animals (3 months old) with either one (*Ins-Cre;Myc+/-*) or both (*Ins-Cre;Myc-/-*) alleles deleted in the β cells. n=3 per group. **p*<0.05, Student's *t* test.

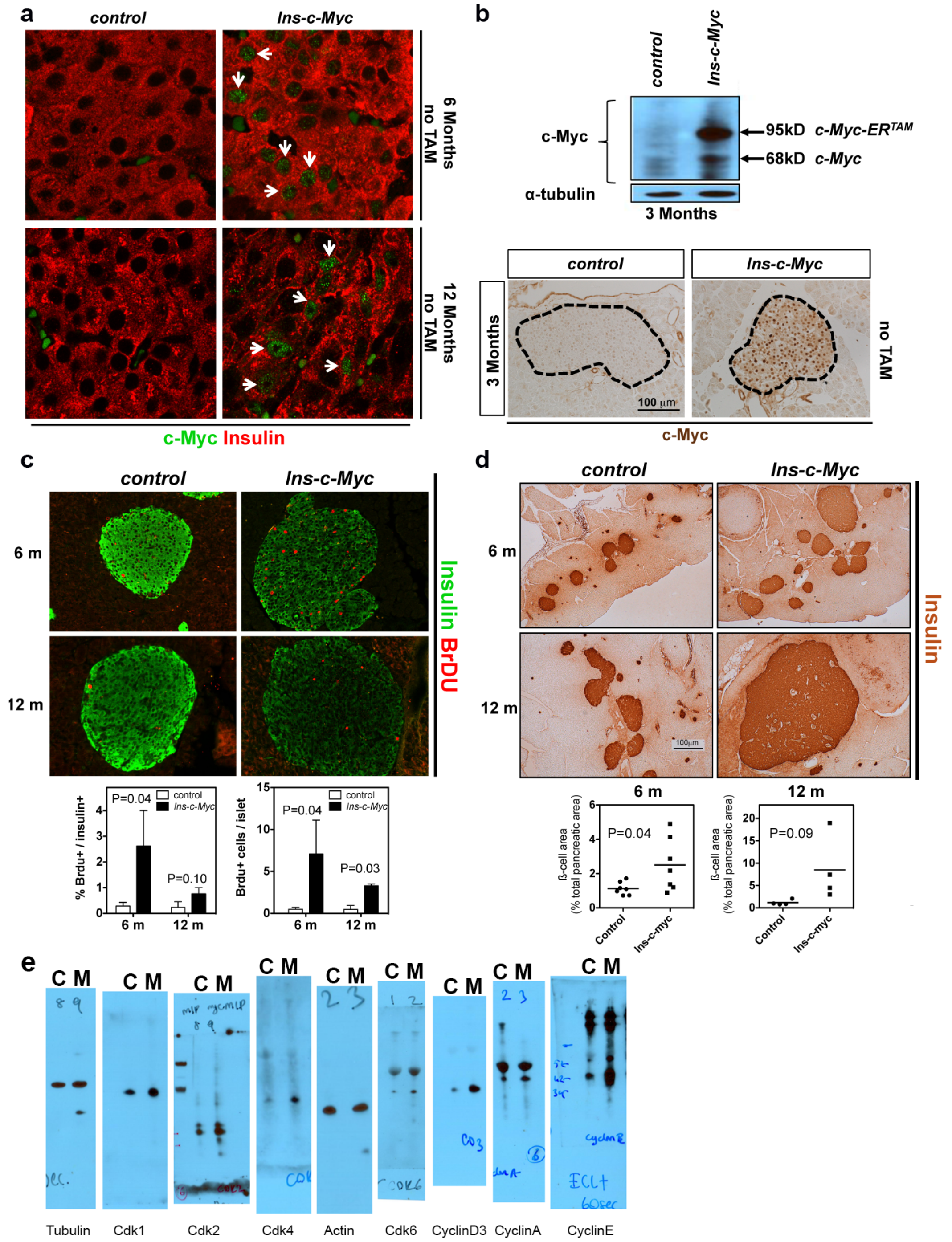

Supplementary Figure 2. **a**. Nuclear accumulation of c-Myc (green) in β cells (red) is readily detectable in animals at 6 and 12 months of age without administration of TAM. **b**. c-Myc-ER^{TAM} fusion protein is detectable by western blotting and immunostaining in islets isolated from 3-month-old transgenic mice. **c**. BrdU incorporation (shown in red) is robust in β cells (shown in green) in older *Ins-c-Myc* transgenic animals**.** Quantification of BrdU incorporation shows increased replicative capacity in β cells in animals as old as one year. 6 months old, control=4, transgenic=4 animals, and 12 months old, control=2, transgenic=3 animals. Error bars indicate ± SD. *P* value calculated using Student's *t* test. **d**. A significant increase in β cell mass occurs through the life of the transgenic animals as compared to control littermates. For quantification of β cell mass, 6 months old, control=7, transgenic=7 animals, and 12 months old, control=4, transgenic=4 animals were used. *P* value calculated using Student's *t* test. **e**. Original western blots shown for cell cycle proteins (Cdk2, Cdk4, Cyclind3, CyclinA and CyclinE), loading controls Tubulin and Actin, and a representative blot shown for Cdk1.

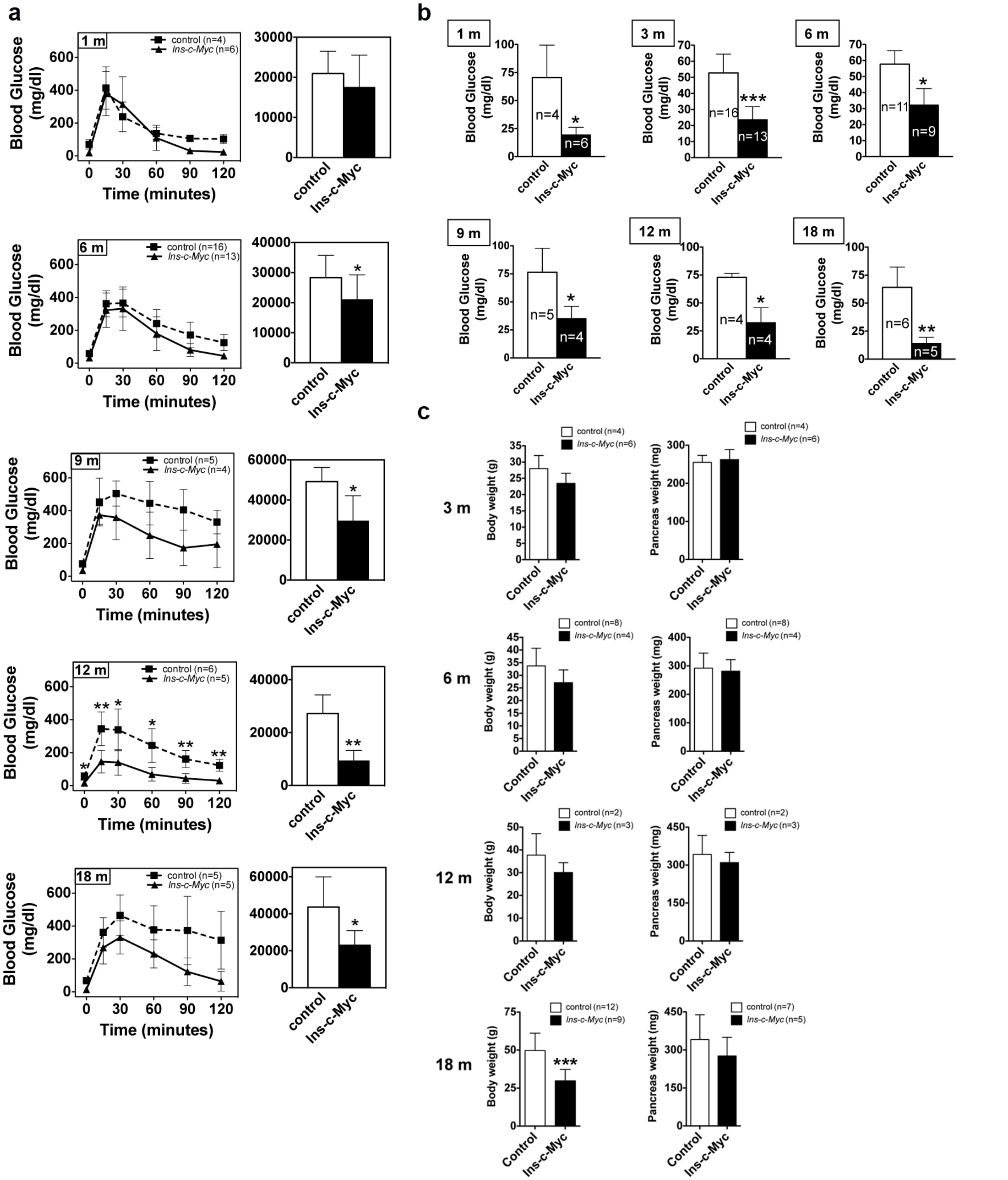

Supplementary Figure 3. **a.** Glucose tolerance tests on mice at the different ages noted in the figure revealed that *Ins-c-Myc* transgenic mice have improved clearance of glucose from the blood throughout their lifespan. The corresponding area under the curve is noted next to the glucose tolerance test. Error bars indicate ± SD. **p*, ≤0.05, ***p*, <0.005, Student's *t* test. **b**. Fasted blood glucose is persistently low in transgenic animals as compared to control littermates in the absence of TAM administration. Error bars indicate ± SD. **p*, ≤0.05, ***p*, <0.005, ****p*, <0.0005, Student's *t* test. **c**. Body weight and pancreas weight measurements for *Ins-c-Myc* transgenic mice and control littermates at the times indicated. Error bars indicate ± SD. **p*, <0.0005, Student's *t* test.

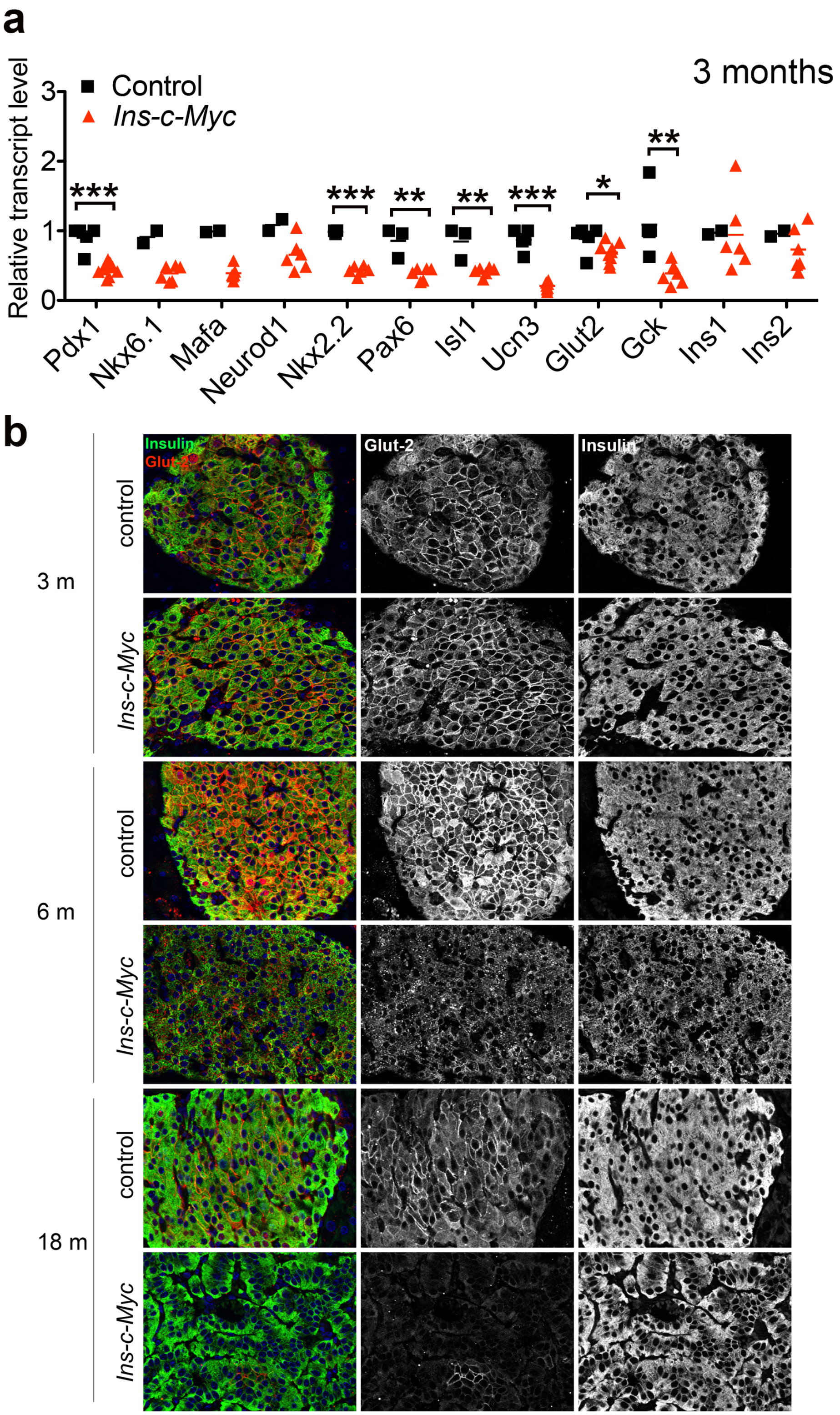

Supplementary Figure 4. **a**. QPCR analysis on islets from three-month-old *Ins-c-Myc* animals showed a reduction in the canonical β cell transcription factors including *Pdx1*, *Nkx6.1*, *Mafa*, *Neurod1, Nkx2.2, Pax6* and *Isl1*. Maturation marker *Ucn3* was also reduced in the transgenic dataset, as was the transporter *Glut-2* and the glucose sensor *Gck*. No significant changes were seen in *Ins1* and *Ins2* expression. Control samples, n=2-5, transgenic samples, n=6-9. *$p<0.05$, **$p<0.005$, ***$p<0.0005$, Student's *t* test. **b**. At three months of age, immunostaining did not reveal changes in the levels of Glut-2. Over time (6 months and 18 months shown), Glut-2 staining was significantly diminished in β cells.

a

b

c

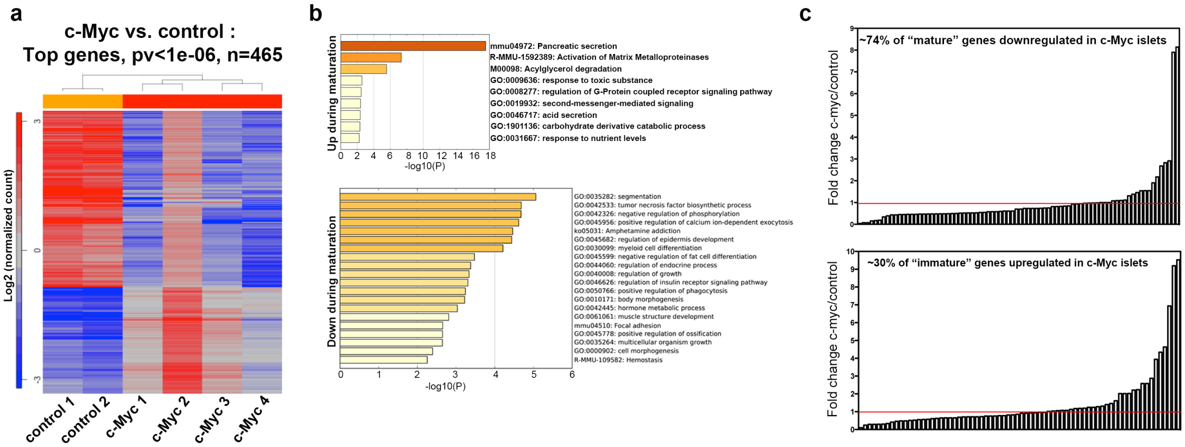

d

geneId	mgi_symbol	description	foldChange_transVsWt	PValue_transVsWt
ENSMUSG00000032902	Slc16a1	solute carrier family 16 (monocarboxylic acid transporters) member 1	3.30351066	9.73E-09
ENSMUSG00000037012	Hk1	hexokinase 1	0.603698449	0.059857
ENSMUSG00000063229	Ldha	lactate dehydrogenase A	0.838161407	0.61137
ENSMUSG00000030934	Oat	ornithine aminotransferase	0.75675594	0.20785
ENSMUSG00000017493	Igfbp4	insulin-like growth factor binding protein 4	0.51666462	0.028341
ENSMUSG00000027187	Cat	catalase	0.857673232	0.49981
ENSMUSG00000030220	Arhgdib	Rho dissociation inhibitor (GDI) beta	1.241599602	0.46068
ENSMUSG00000021127	Zfp36l1	zinc finger protein 36 type-like 1	0.701395461	0.11228
ENSMUSG00000061353	Cxcl12	chemokine (C-X-C motif) ligand 12	0.713309454	0.083735
ENSMUSG00000025780	Itih5	inter-alpha (globulin) inhibitor H5	0.48727226	0.00044399
ENSMUSG00000029231	Pdgfra	platelet derived growth factor receptorha polypeptide	0.273175499	8.37E-06
ENSMUSG00000028266	Lmo4	LIM domain only 4	0.980371194	0.91676
ENSMUSG00000029860	Zyx	zyxin	0.611998491	0.04569
ENSMUSG00000032402	Smad3	SMAD family member 3	1.236446657	0.57439
ENSMUSG00000000628	Hk2	hexokinase 2	1.494952867	0.56271
ENSMUSG00000025877	Hk3	hexokinase 3	3.740937855	0.07356

e

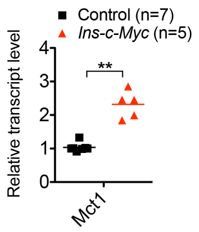

f

geneId	mgi_symbol	description	foldChange_transVsWt	PValue_transVsWt
ENSMUSG00000019942	**<u>Cdk1</u>**	cyclin-dependent kinase 1	3.467509896	0.0072493
ENSMUSG00000006728	**<u>Cdk4</u>**	cyclin-dependent kinase 4	1.69313851	0.00086708
ENSMUSG00000040274	**<u>Cdk6</u>**	cyclin-dependent kinase 6	1.735197645	0.13858
ENSMUSG00000027793	**<u>Ccna1</u>**	cyclin A1	10.40857022	0.41733
ENSMUSG00000002068	**<u>Ccne1</u>**	cyclin E1	2.11608855	0.0060606
ENSMUSG00000027715	Ccna2	cyclin A2	2.107452333	0.010165
ENSMUSG00000032218	Ccnb2	cyclin B2	2.643776098	0.0083325
ENSMUSG00000026683	Nuf2	NUF280 kinetochore complex componentolog (S. cerevisiae)	2.941801698	0.0077775
ENSMUSG00000023505	Cdca3	cell division cycle associated 3	2.701199134	0.0073919
ENSMUSG00000028873	Cdca8	cell division cycle associated 8	2.336420584	0.013467
ENSMUSG00000028678	Kif2c	kinesin family member 2C	6.671448351	4.05E-05
ENSMUSG00000079553	Kifc1	kinesin family member C1	2.854820426	0.0044954
ENSMUSG00000005233	Spc25	SPC2580 kinetochore complex componentolog (S. cerevisiae)	1.838335391	0.0031565
ENSMUSG00000001403	Ube2c	ubiquitin-conjugating enzyme E2C	2.614255233	0.016997

g

geneId	mgi_symbol	description	foldChange_transVsWt	PValue_transVsWt
ENSMUSG00000062647	Rpl7a	ribosomal protein L7A	2.784464694	5.84E-08
ENSMUSG00000062006	Rpl34	ribosomal protein L34	4.34182025	4.85E-06
ENSMUSG00000007892	Rplp1	ribosomal proteinge, P1	4.549790133	1.14E-08
ENSMUSG00000032399	Rpl4	ribosomal protein L4	3.420719956	1.37E-07
ENSMUSG00000066362	Rps13-ps1	ribosomal protein S13udogene 1	3.564994868	4.61E-12
ENSMUSG00000006333	Rps9	ribosomal protein S9	2.999454195	5.04E-11
ENSMUSG00000060036	Rpl3	ribosomal protein L3	3.584072944	8.22E-07
ENSMUSG00000029614	Rpl6	ribosomal protein L6	3.963019318	1.59E-07
ENSMUSG00000025794	Rpl14	ribosomal protein L14	3.14792245	1.31E-12
ENSMUSG00000000740	Rpl13	ribosomal protein L13	3.414797433	2.51E-08
ENSMUSG00000030432	Rpl28	ribosomal protein L28	2.799947934	1.61E-09
ENSMUSG00000048758	Rpl29	ribosomal protein L29	3.797368484	3.13E-07
ENSMUSG00000069682	Gm10275	predicted pseudogene 10275	5.314743256	1.08E-08
ENSMUSG00000025362	Rps26	ribosomal protein S26	4.284420256	1.41E-07
ENSMUSG00000025508	Rplp2	ribosomal proteinge P2	2.544355042	6.74E-09
ENSMUSG00000063457	Rps15	ribosomal protein S15	3.056753042	7.72E-09
ENSMUSG00000052146	Rps10	ribosomal protein S10	3.556849669	1.56E-08
ENSMUSG00000047675	Rps8	ribosomal protein S8	3.120765598	3.99E-07
ENSMUSG00000008668	Rps18	ribosomal protein S18	4.519928653	9.24E-08
ENSMUSG00000003429	Rps11	ribosomal protein S11	2.306491539	5.44E-07
ENSMUSG00000037805	Rpl10a	ribosomal protein L10A	3.922300312	5.84E-09
ENSMUSG00000044533	Rps2	ribosomal protein S2	3.247433539	7.63E-13
ENSMUSG00000062328	Rpl17	ribosomal protein L17	3.431882122	9.61E-07
ENSMUSG00000059070	Rpl18	ribosomal protein L18	3.13116601	4.21E-07
ENSMUSG00000057863	Rpl36	ribosomal protein L36	3.248108894	5.83E-08
ENSMUSG00000003970	Rpl8	ribosomal protein L8	2.533619717	5.83E-09
ENSMUSG00000068240	Gm11808	predicted gene 11808	3.676415944	6.47E-10

Supplementary Figure 5. **a**. Heat map of top differentially expressed genes (p value < 1e-06) between transgenic (*Ins-c-Myc*) and control islets of log2 normalized RNA-seq count data. **b**. Gene ontology (GO) analysis of genes differentially expressed in mature β cells as compared to postnatal islets (P1) from Blum et al. **c**. Analysis of genes differentially expressed in mature β cells as compared to postnatal islets (P1) reveals that the majority of "mature" genes are downregulated in c-Myc β cells. Conversely, ~30% of "immature" genes are upregulated in c-Myc β cells. **d**. Expression levels of disallowed genes in β cells with stabilized c-Myc reveal changes in *Mct1* (*Slc16a1*), *HKII* and *HKIII* (shown in red). **e**. QPCR analysis validates the increased expression of *Mct1* in *Ins-c-Myc* islets (n=5) relative to controls (n=7) at three months of age. **p<0.005, Student's t test. **f**. Expression level of cell cycle genes is consistently upregulated in c-Myc β cells. Genes validated at the protein level are in bold and underlined. **g**. A global increase in ribosomal proteins (fold change of transgenic over controls shown in green) was detected in islets isolated from *Ins-c-Myc* animals as compared to control littermates. "logFC" means log2 fold change.

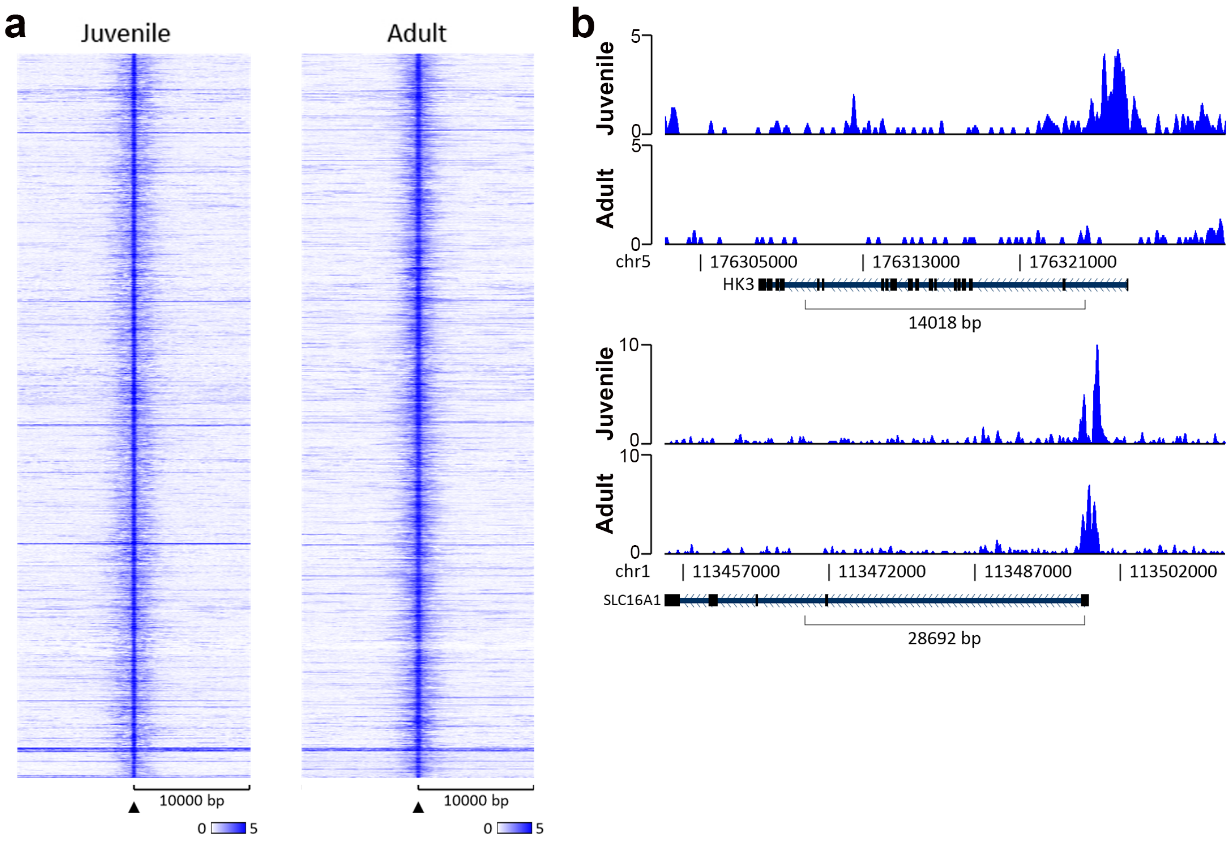

Supplementary Figure 6. **a**. Chromatin immunoprecipitation data from juvenile (5 years) and adult (48 years) donors showing the overall landscape of H3K4 trimethylation. Heatmap shows enrichment of H3K4Me3 in juvenile and adult islet datasets. **b**. H3K4 trimethylation is increased in the juvenile (5 years) sample as compared to adult (48 years) at the start sites of the disallowed genes *HK3* and *MCT1*.

Supplementary Table 1. Primer sequences used for QPCR and chromatin immunoprecipitation analyses.

**Mouse specific primers**		**Sequence**
Pdx1	Forward	CGGCTGAGCAAGCTAAGGTT
	Reverse	GGAAGAAGCGCTCTCTTTGAAA
Nkx6.1	Forward	TCAGGTCAAGGTCTGGTTCCA
	Reverse	CGGTCTCCGAGTCCTGCTT
Neurod1	Forward	TCCGGTGCCGCTGC
	Reverse	GCGAATGGCTATCGAAAGACA
Mafa	Forward	GCTGGTATCCATGTCCGTGC
	Reverse	TGTTTCAGTCGGATGACCTCC
Ucn3	Forward	AAGCCTCTCCCACAAGTTCTA
	Reverse	GAGGTGCGTTTGGTTGTCATC
Ccna2	Forward	ACCACTGACACCTCTTGACTAT
	Reverse	GGTGATTCAAAACTGCCATCCA
Pc1/3	Forward	AGGCAGCTGGCGTGTTTG
	Reverse	GAAGCTGGTTCCGCTTGGA
c-Myc	Forward	GCTCTCCATCCTATGTTGCGG
	Reverse	TCCAAGTAACTCGGTCATCATCT
Ki67	Forward	CAAGGCGAGCCTCAAGAGATA
	Reverse	TGTGCTGTTCTACATGCCCTG
Pcna	Forward	ATGCCGTCGGGTGAATTTG
	Reverse	TCTCCAATGTGGCTAAGGTCTC
Cdk1	Forward	AGGTACTTACGGTGTGGTGTAT
	Reverse	CTCGCTTTCAAGTCTGATCTTCT
Cdk2	Forward	CAAAAACAAGTTGACGGGAGAAG
	Reverse	CAGTCTCAGTGTCGAGCCG
Cdk4	Forward	TCAGCACAGTTCGTGAGGTG
	Reverse	TCAGCCGTACAACATTGGGAT
Cdk6	Forward	CCTTACCTCGGTGGTCGTC
	Reverse	GAACTTCCACGAAAAAGAGGCT
Nkx2.2	Forward	AAGCATTTCAAAACCGACGGA
	Reverse	CCTCAAATCCACAGATGACCAGA
Pax6	Forward	GGATCCGGAGGCTGCC
	Reverse	CCAAGCTGATTCACTCCGCT
Isl1	Forward	ATGATGGTGGTTTACAGGCTAAC
	Reverse	TCGATGCTACTTCACTGCCAG
Glut2	Forward	AGTGGGCGGAATGGTCG
	Reverse	TGCTTTGATCCTTCCAAGTTTGT
Gck	Forward	TGCCCACCTACGTGCGT
	Reverse	TCCCAGGTCTAAAGGAGAGAAAGTC
Ins1	Forward	CTGCTGGCCCTGCTTGC
	Reverse	GGGTCGAGGTGGGCCTT
Ins2	Forward	CCTGCTGGCCCTGCTCTT
	Reverse	GGCTGGGTAGTGGTGGGTCTA
Mct1	Forward	TGTTAGTCGGAGCCTTCATTTC
	Reverse	CACTGGTCGTTGCACTGAATA
CyclophilinA	Forward	TCACAGAATTATTCCAGGATTCATG
	Reverse	TGCCGCCAGTGCCATT
**Rat specific primers**		
Pdx1	Forward	CCCAGCCGCGTTCATCT
	Reverse	CCACGCGTGAGCTTTGGT
Nkx6.1	Forward	GCGCGCCTTGCCTGTA
	Reverse	TCTTCCCATCTTTGTCCAACAA

Mafa	Forward	AGGAGGTCATCCGACTGAAACA
	Reverse	GCGTAGCCGCGGTTCTT
Hnf4a	Forward	TGCAGGCAGAGGTCCTGTCT
	Reverse	TCGCCATTGATCCCAGAGA
Pax4	Forward	ATGCAGCAGGACGGTCTCA
	Reverse	GGCCGGCCATTCACAA
Pax6	Forward	CAGCCCACCACACCTGTCT
	Reverse	GTCTGTGCGGCCCAACAT
c-Myc	Forward	TGTATGTGGAGCGGCTTCTC
	Reverse	CCTGGTAAGAGGCCAGCTTC
CyclophilinA	Forward	GGTGAAAGAAGGCATGAGCAT
	Reverse	GCCATTCCTGGACCCAAAA
**QPCR primers for ChIP**		
Pcsk1 (-1719)	Forward	CACCCAACATTGTGTTCTGG
	Reverse	GTGCATGGCATAAAGCAAGA
Pcsk1 (-5861)	Forward	ATGGTGGAATTGCAAAATGG
	Reverse	TGGGAAGTTTCCAGCTTCTG
Pdx1 (-193)	Forward	GGATCAGGCGACTGAGAGAG
	Reverse	ACTGCTCTCCTGGGGAACC
Neurod1 (-375)	Forward	GTCCGCGGAGTCTCTAACTG
	Reverse	GAACCACGTGACCTGCCTAT
Neurod1 (-3838)	Forward	CACATCAACAACCATGCACA
	Reverse	AATAAACGCTGCCAGAGCAC
Neurod1 (-4838)	Forward	GCTTGCTCTTCCCAGAATCA
	Reverse	CCAATCAATTCTCCAGCTGTT
Ins2 (-1562)	Forward	TCTCATGGGGGAGAGAAATG
	Reverse	CGTCTCCATGAGGAATGACA
Ins2 (-6387)	Forward	GCTGGGCAGGAGAGACATAG
	Reverse	AATTAGGCAGGCCTCAGGAT
