# Supplementary material for: Replication confers β cell immaturity
Source: Nat Commun. 2018 Feb 2;9:485. doi: 10.1038/s41467-018-02939-0 (PMC5797102; doi:10.1038/s41467-018-02939-0)
Supplement: Supplementary file 3 — Description of Additional Supplementary Files [file 41467_2018_2939_MOESM3_ESM.pdf]

Description of Additional Supplementary Files

File Name: Supplementary Data 1
Description: Comparison of gene expression after RNA-seq analysis of islets isolated from Ins-c-Myc transgenic islets as well as littermate controls revealed a downregulation of 290 RNAs (red) and upregulation of 175 RNAs (green) in the Ins-c-Myc islets using a nominal p value cutoff at 10-6.
